# Fano resonance in anodic aluminum oxide based photonic crystals

**DOI:** 10.1038/srep03601

**Published:** 2014-01-08

**Authors:** Guo Liang Shang, Guang Tao Fei, Yao Zhang, Peng Yan, Shao Hui Xu, Hao Miao Ouyang, Li De Zhang

**Affiliations:** 1 Key Laboratory of Materials Physics and Anhui Key Laboratory of Nanomaterials and Nanostructures, Institute of Solid State Physics, Hefei Institutes of Physical Science, Chinese Academy of Sciences, P. O. Box 1129, Hefei, 230031, P. R. China; 2 Hefei National Laboratory for Physical Sciences at the Microscale, University of Science and Technology of China, Hefei, Anhui 230026, P. R. China

## Abstract

Anodic aluminum oxide based photonic crystals with periodic porous structure have been prepared using voltage compensation method. The as-prepared sample showed an ultra-narrow photonic bandgap. Asymmetric line-shape profiles of the photonic bandgaps have been observed, which is attributed to Fano resonance between the photonic bandgap state of photonic crystal and continuum scattering state of porous structure. And the exhibited Fano resonance shows more clearly when the sample is saturated ethanol gas than air-filled. Further theoretical analysis by transfer matrix method verified these results. These findings provide a better understanding on the nature of photonic bandgaps of photonic crystals made up of porous materials, in which the porous structures not only exist as layers of effective-refractive-index material providing Bragg scattering, but also provide a continuum light scattering state to interact with Bragg scattering state to show an asymmetric line-shape profile.

Fano resonance^1^ is a type of resonant scattering phenomenon resulted from interference of a continuum state and a discrete state, which will give rise to an asymmetric line-shape of the profile^2^,^3^,^4^,^5^,^6^, and has been found in many fields of physics^6^,^7^,^8^,^9^,^10^,^11^,^12^. Fano resonance as one of the important scattering phenomena will play an important role in the future integrated optical systems in which scatterings from different factors coexist^5^. Photonic crystals (PCs)^13^,^14^, as novel optical materials with different refractive index periodically stacked in space, can exhibit photonic bandgaps (PBGs) in which propagation of incident light is inhibited. Since PCs exhibit a wide range of application prospects, the study of Fano resonance in PCs has become of great importance and drawn many attentions^15^,^16^,^17^,^18^,^19^,^20^,^21^,^22^,^23^. Recently, M.V. Rybin *et al.*^24^,^25^*.* observed Fano resonance owing to the interaction between continuum Mie scattering and a narrow Bragg Band in the PBG of the synthetic opal photonic crystals. I.V. Soboleva *et al.*^26^*.* analyzed Fano resonances in 1D photonic crystals of ZrO_2_/SiO_2_ bilayers multilayer structures. S. Nojima *et al.*^27^*.* used Fano resonances to elucidate the resonance nature of the localized defect states in photonic crystals. Furthermore, it was found that the asymmetry PBG profiles can be tuned by changing the effective dielectric constant of materials^24^, light incident angle^28^ and thickness of the PCs^26^.

Anodic Aluminum Oxide (AAO) based PC is a kind of porous material containing alternative stacked stem channel layer and branched channel layer^29^,^30^,^31^,^32^,^33^. Strong light scattering caused by the porous structure of AAO could serve as a continuum state. Meanwhile, Bragg scattering caused by periodically layered structure in AAO can be regarded as a discrete state. So the interference may occur between these two states^5^, in this case Fano asymmetry profile in the PBG of AAO based PC could be observed. However little information is available about Fano resonance in AAO based PC. Although the asymmetry profiles of PBGs have been reported^29^,^30^,^31^,^32^, they are not regarded as the results of Fano resonance. Mostly, the asymmetry profile of PBG is attributed to inhomogeneous structure of the sample and the resulted superposition of multiple PCs. Therefore, preparation of photonic crystal with homogeneous structure is very necessary for the study of Fano resonance in AAO based PCs.

In this paper, we prepared the AAO based PC with narrow photonic bandgaps using compensation voltage oxidation mode^33^,^34^, which effectively avoids the undesired asymmetry from tapered nanopores and resulted nonperiodicity. In this AAO based PC with narrow PBG, we observed Fano resonance phenomenon of which the origin can be determined as interference of the continuum light scattering state and discrete light scattering state.

## Results

### Morphology and bandgap profile

**Figure 1:**
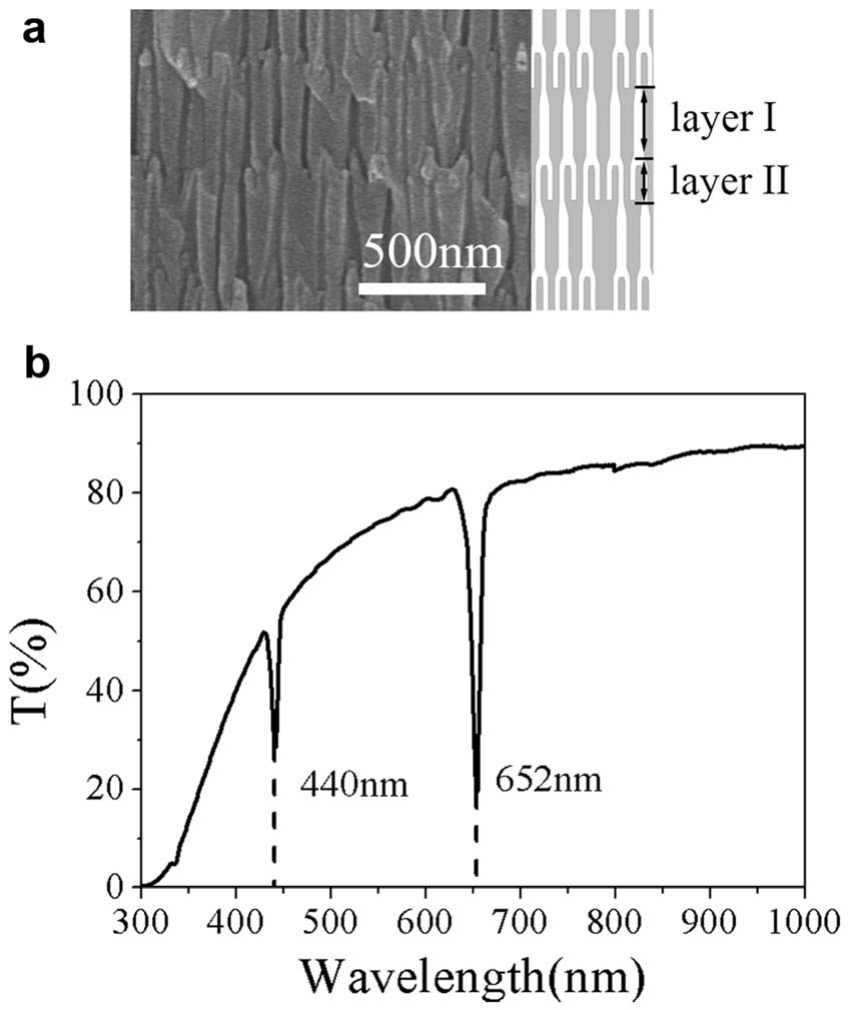
SEM images and Transmittance of the as-prepared PC. (a) Periodic structure of the AAO based PC (left), and schematic illustration (right) in which the white part is air pore and the gray part is alumina. (b) Transmittance spectrum of the as-prepared AAO based PC.

Figure 1 shows the cross-sectional SEM images and transmittance spectrum of the AAO based PC. The as-prepared PC was about 47 μm thick (Figure 1a). The periodic structure composes of alternately stacked stem channel layers (layer I) and branched channel layers (layer II), leading to a quasi-one dimensional PC along the pores growth direction. The layered structure was schematic illustrated in the right side of Figure 1a, in which the white part is air pore and the gray part is alumina. The thicknesses of layer I and layer II are about 320 and 140 nm, and corresponding pore diameters are of 50 and 25 nm, respectively.

Figure 1b is transmittance spectrum of the as-prepared sample, which can be considered as two composing parts: One is the narrow PBGs with centers located at 440 and 652 nm, respectively. The corresponding full width at half maximum (FWHM) of the PBGs are 8 and 10 nm, respectively. These ultra-narrow PBGs are benefited from the uniform pore structure from beginning to end (see Supplementary Figure S1) generated by the applied compensation voltage mode during preparation which avoids the undesired asymmetry from tapered nanopores and resulted nonperiodicity^33^,^34^. The other is the continually decreased transmittance of the spectrum (CDTS) with decreasing wavelength, which reduces to zero when the wavelength is below 300 nm. Since AAO based PC is a kind of porous material with the pore diameter is in nanometer order of magnitude and thicknesses of stem and branched channel layers are all around several hundred nanometers, the light scattering will be stronger in shorter wavelength region, therefore, the transmittance in shorter wavelength is much lower than that in longer wavelength.

This narrow PBG will be great beneficial to demonstrate various physical phenomena resulted from different factors in PCs. Generally, PBG of an ideal PC exhibits a symmetry profile if only strong Bragg scattering is considered. However, owing to the existence of light scattering from porous structure, interference between this CDTS and Bragg scattering might occur^5^, a Fano type profile of an asymmetric band in spectrum would be expected.

**Figure 2:**
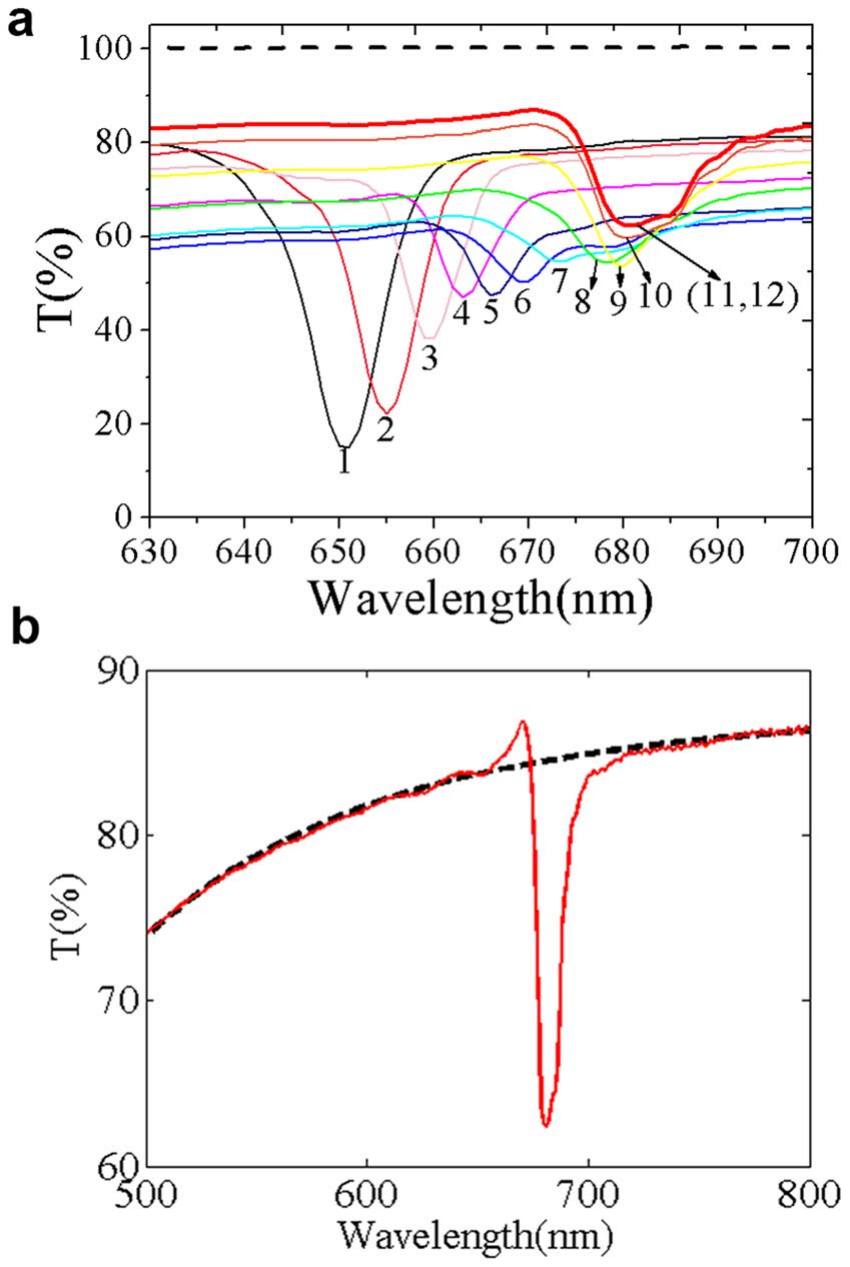
PBG changes with time and exhibited an asymmetry profile of asymmetry. (a) PBGs changes with time at an interval of 90 s when they continually adsorbed ethanol gas, dash line is transmittance spectrum of saturated ethanol gas. And, asymmetric profile exists in the PBGs, such as line 11 and 12 (b) shows a clearly asymmetry profile in which the short wavelength photonic band edge is much higher than the long wavelength. Dash line is the fitting curve without PBG.

Figure 2 shows the change of transmittance spectra of the as-prepared sample with exposing time at an interval of 90 s to a saturated ethanol gas at room temperature. With the increase of time for the sample placed in the surrounding of saturated ethanol gas, the adsorbed ethanol gas in the pores will gradually increase. It can be seen from Figure 2a that the position of PBG has a red shift with the increase of adsorbed ethanol gas in the pores of AAO; meanwhile, transmittance at bottom of the band gap has an upward trend over time. After 11 times measurement (about 16.5 minutes), the PC reaches adsorption equilibrium which can be verified by the coincidence of curves 11 and 12. It is noted that, light absorption of ethanol has little impact on the PBG profile located in visible light region, which is demonstrated by the transmittance spectrum of saturated ethanol gas shown by the dash line in Figure 2a.

Furthermore, transmittance spectra (Figure 2a) exhibit asymmetry profiles which should be drawn particular attention. It can be seen that for curve 1, the photonic band edge (PBE) in short wavelength side is relatively flat, and the PBE in long wavelength side drops abruptly. However, for curves 11 and 12 the transmittance spectrum shows different type of asymmetry profiles: The short wavelength PBE drops abruptly while the long wavelength PBE is relatively flat. By comparing the transmittance spectrum of curve 11 in Figure 2a with the fitting curve without PBG shown by Figure 2b, this asymmetry can be seen clearly. Meanwhile, the short wavelength PBE shows a dramatically increase. These results indicate that it has characteristics of Fano resonance, so we think the exhibited asymmetry profile of the PBGs results from resonance between the Bragg scattering and CDTS.

### Theoretical simulations

In order to understand the physical nature of asymmetry profile of PBGs, theoretical simulations were carried out. The effective dielectric constant of stem channel layer and branched channel layer can be given by the following Bruggeman formula^35^: 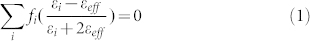
where *ε_i_* is dielectric constant of *ith* substance, *f_i_* is the corresponding volume fraction, *ε_eff_* is effective dielectric constant of the mixture.

The transmission (*t_1_*) and reflection (*r_1_*) parts of one period (layer I and layer II shown in Figure 1a) were first calculated based on finite element analysis method, in which strong light scattering by the porous structure and Bragg scattering were both taken into account. Then, the PBG profiles were analyzed using Transfer Matrix Method (TMM). The transfer matrix (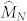) of PC with *N* periods can be written as: 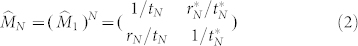
where 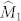 is the transfer matrix of the sample within one period, *t_N_* and *r_N_* are the transmission and reflection part of PC with *N* periods, respectively. Superscript star (*) stands for conjugation of the corresponding terms. Herein, we have |*t_N_*|^2^ + |*r_N_*|^2^ = *T_N_* + *R_N_* = 1 for lossless medium, in which *T_N_* and *R_N_* are the transmittance and reflectance, respectively.

Simplified structure with a stem and a branched channel layer in one period as shown in Figure 1a is adopted in the simulations. The simulated thicknesses of layer I and layer II are 320 and 140 nm, respectively, and the corresponding pore diameters of these layers are 50 and 25 nm, respectively. The refractive indexes are *n*_alumina_ = 1.7, *n*_air_ = 1, *n*_ethanol_ = 1.36 for alumina, air and ethanol, respectively. Thus, the corresponding dielectric constants (*ε* = *n*^2^) are *ε*_alumina_ = 2.89, *ε*_air_ = 1, *ε*_ethanol_ = 1.85, for alumina, air and ethanol, respectively. The simulation wavelength ranges from 300 to 1000 nm, and the period number is *N* = 60. In the simulations, strong light scattering by the porous structure and Bragg scattering are both taken into account.

**Figure 3:**
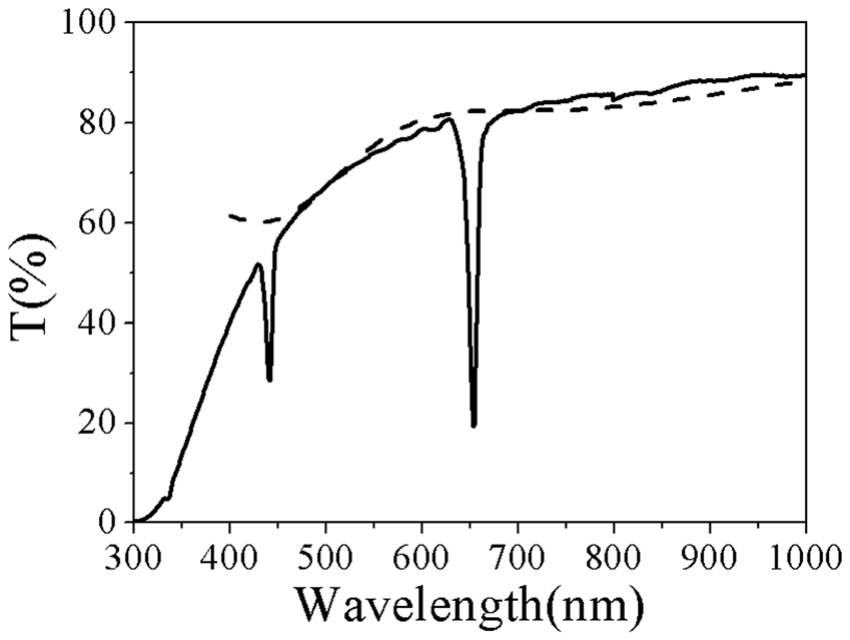
Transmittance decrease from light scattering by the porous structure. Simulated transmittance decreases from light scattering by porous structure as the dash line shown compared with experimental one (solid line).

Figure 3 shows the simulated spectrum only considering transmittance decrease by background light scattering without Bragg scattering in comparison with the experimental result. It can be seen that the simulated transmittance is generally consistent with experimental result when the wavelength is above 450 nm. However, when the wavelength is lower than 450 nm, the simulated transmittance starts to go up which is different from the experimental result. This difference is thought to be that alumina has light absorption when light wavelength is below 450 nm. Comparing the simulated and experimental spectra, the transmittance decrease with decreasing wavelength above wavelength 450 nm is mainly from light scattering by the porous structure. Transmittance spectrum of the as-prepared sample shows that porous AAO based PC not only exist as an effective-refractive-index material leading to the photonic bandgaps, but also generate a strong light scattering shown by the decrease of the transmittance with the decreasing wavelength.

**Figure 4:**
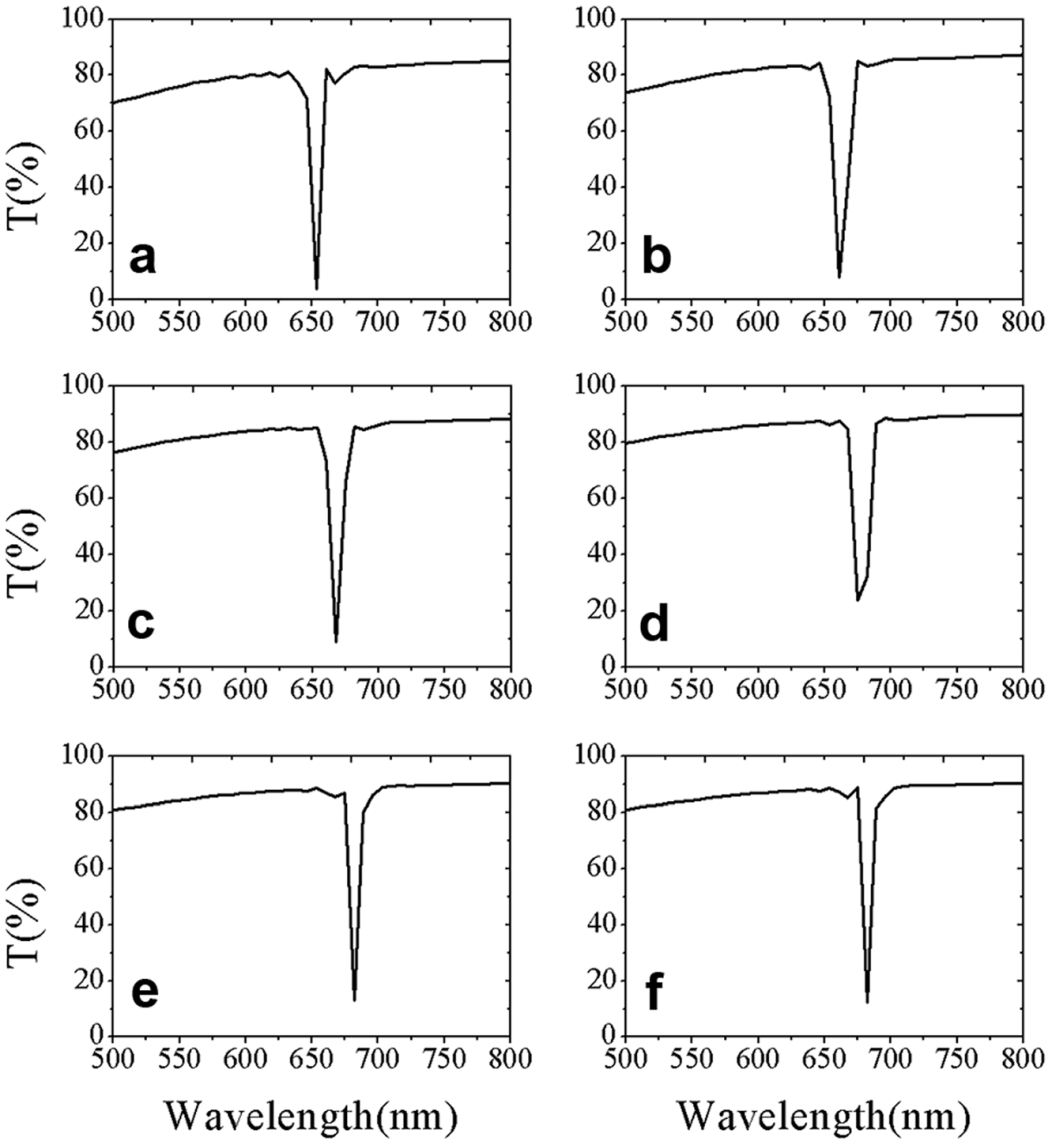
Simulation results of the PBG at different wavelengths. Simulations show that the PBG exhibits a Fano type profile. Filling refractive indexes of the air pore for image (a–f) are 1.0000, 1.0600, 1.1030, 1.1575, 1.1740 and 1.1760, respectively.

Furthermore, we introduced the Bragg interference by alternated dielectric layers in simulations. Refractive indexes of the pores were chosen to be 1.0000, 1.0600, 1.1030, 1.1575, 1.1740 and 1.1760, respectively, and the results are shown in Figure 4. It can be seen that with the increase of refractive index of filler in the channels, the position of PBG has a red shift, in accordance with the experimental results. And the transmittance spectra show different asymmetry profiles when the fillers in the pores has different refractive index. When the filling refractive index in pores is 1.0000 for curve in Figure 4a, the PBE in short wavelength side is relatively flat and the PBE in long wavelength side drops abruptly; however, when the filling refractive index in pores is 1.1760 for curve in Figure 4f, the short wavelength PBE drops abruptly while the long wavelength PBE is relatively flat.

**Figure 5:**
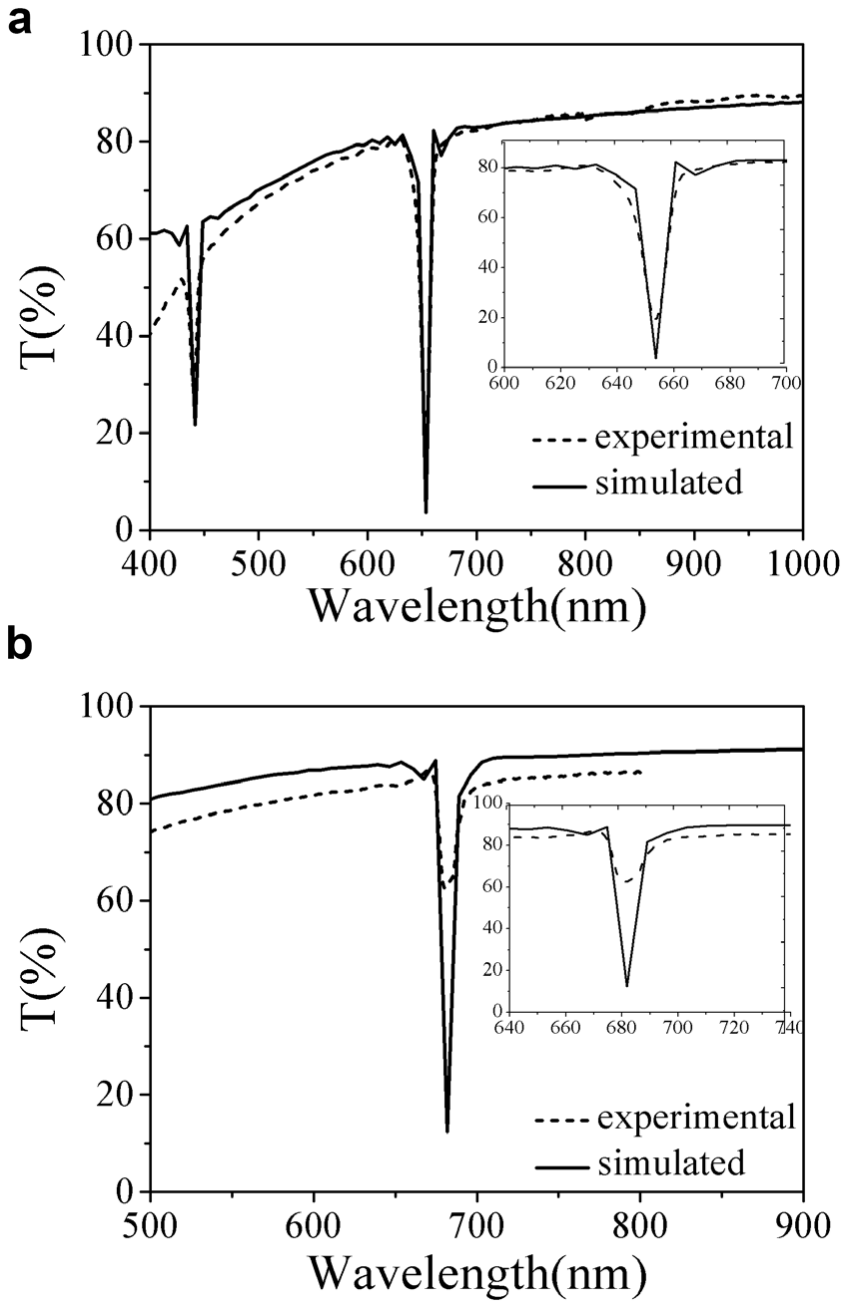
Simulated results compared with the experimental one. Compare the simulation (solid lines) and experimental (dash lines) spectra when the as-prepared sample adsorbs no ethanol gas (a) and saturated ethanol gas (b), respectively. Inset is the corresponding enlarged spectrum.

For better comparison, the simulated results and experimental ones are plotted in Figure 5, where figure 5a is for the sample of air-filled and figure 5b is for that of ethanol gas saturated. As we can see from the simulated spectrum, the PBG is located at 652 nm which is consistent with the experimental one of air-filled. Meanwhile, the short wavelength PBE was relatively flat, but the long wavelength PBE was drop abruptly. Figure 5b shows the simulated curve in Figure 4f and experimental curve 11 in Figure 2a. It can be seen that the simulated PBG located at 681 nm which consists with the experimental PBG with the equilibrium adsorption of saturated ethanol gas, of which the long wavelength PBE was relatively flat; however, the short wavelength PBE was drop abruptly. Comparing these simulations with the experiment results, we can attribute the asymmetry profile in our experiments to the interference of the Bragg scattering by periodically layered structure and strong scattering by porous structure, which is Fano resonance.

### Fano asymmetry parameter

Generally, Fano resonance can be described by factor F(Ω)^1^, which can be expressed by the following equation (Eq. 3): 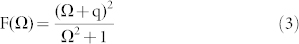
where q is Fano asymmetry parameter, which describes the degree of asymmetry, Ω = (*ω* − *ω*_o_)/(*γ*/2) is dimensionless frequency, *ω*_o_ is a central frequency and *γ* is width of the narrow band, in our experiment we take the FWHM of PBG as value *γ*.

**Figure 6:**
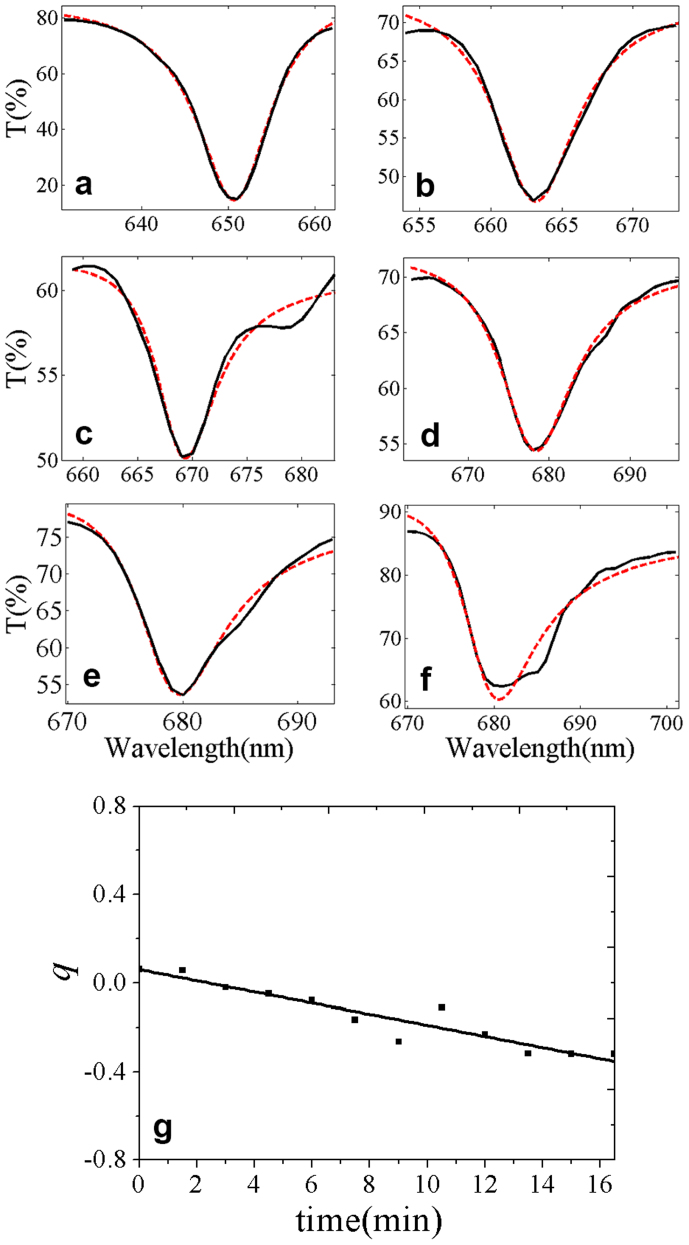
Different Fano asymmetry parameter q for PBGs. Plots (a–f) are PBGs of curves 1, 4, 6, 8, 9 and 11 in Figure 2a, respectively, and the dash lines are fitting profiles based on Eq. 3 from which the corresponding Fano asymmetry parameter q is obtained. Figure 6g is parameter q change with time.

Figure 6a–f shows the fitting curves with Eq. 3 for the experimental transmittance spectra curves 1, 4, 6, 8, 9 and 11 in Figure 2a, respectively. Figure 6g is the relationship between asymmetry parameter q obtained from Figure 6a–f and exposing time. As we can see from Figure 6g, with time increase, adsorbed ethanol gas in the pores will gradually increase, the asymmetry parameter q changes from positive value to negative one. Meanwhile, the absolute value of q which means asymmetry strength of the profile is larger for curves 11 and 12 than that for curve 1. That is to say, the corresponding Fano resonance is stronger. This indicates that the absorbed ethanol gas in the pores affects Fano resonance intensity.

## Discussion

Using compensation voltage mode, alumina PC with narrow PBG was prepared, which could avoid the bandgap profile asymmetry from the superposition of PBGs caused by structural inconsistencies. We found Fano resonance in the as-prepared alumina PC with narrow PBG, and this kind of resonance shows an asymmetric profile of the PBG. Transmittance spectra of the PC showed different asymmetry profiles with different asymmetry parameters by changing the effective refractive index of pores.

The Fano resonance could attribute to the interference between PBGs and background scattering. In the as-prepared samples there are two types of scattering states. One is Bragg scattering which can be regarded as a discrete state, and the other is the strong scattering revealed by decrease background of the transmittance which can be seen as a continuum state. Interference between the discrete state and the continuum state gives rise to a Fano resonance showing asymmetry profiles. According to the experimental results, we believe that the air-filled AAO based PC may have relatively small light scattering ability so that interference between this continuum light scattering and Bragg scattering is small. However, the exhibited Fano resonance is more clearly when the AAO based PC sufficiently absorbed saturated ethanol gas than air-filled AAO based PC. Since the transmittance of saturated ethanol gas almost keeps nearly 100% in visible light region (Figure 2a), we think that ethanol molecules has little impact on Fano resonance. Since there may exist capillary condensation of ethanol gas in the pores of AAO, light scattering would include scatterings both from porous structure and capillary condensed ethanol, which should be stronger than light scattering of air-filled PC. So, the interference between this continuum light scattering and Bragg scattering will be stronger and more clearly when the AAO was saturated ethanol gas.

In conclusion, we demonstrated a viable simple method to induce and analyze the Fano resonance in the AAO based PC. These findings could be applied in the fields of design and preparation of PCs with better performance, and some situations in which interaction between photonic bandgaps and scatterings from other factors is not negligible. In addition, using interactions of different scattering states in photonic crystals will provide a possible approach to fine adjustment of band gap structure.

## Methods

### Synthesis the narrow PBG

In our experiment, a two-step oxidation process was used to fabricate the AAO based PC. The pre-treatment and the first oxidation of the AAO template were performed following the method description in our previous papers^33^,^34^. In detail, high-purity aluminum foils (99.999%) were first degreased in acetone and ethanol, then annealed at 500°C under vacuum ambient (about 2 × 10^−5^ Torr) for 5 h. After that, the aluminum foils were electro-polished in a 1:9 volume mixture of HClO_4_ and C_2_H_5_OH for about 3 min. The first oxidation was carried out at a constant voltage of 53 V in 0.3 M/L H_2_C_2_O_4_ for 3 h in ice water bath, then the formed alumina layer was removed by a mixed solution of 6 wt.% phosphoric acid and 1.5 wt.% chromic acid at 60°C. During the second oxidation, the compensation voltage mode was adopted^33^. In detail, in the first period of the voltage waveform, the voltage increased from *V_L_* ( = 23 V) to *V_H_* ( = 53 V) by a quarter of sinusoidal wave within *t* = 30 s and then linearly decreased to *V_L_* from *V_H_* within *t* = 3 min. From the second period, a compensation voltage was introduced. The voltage value of each period is overall higher than the previous one with a constant voltage of 0.055 V. All the second oxidations were controlled by computer and carried out in a water tank with constant temperature of 16°C.

### Fano resonance characterization

After the sample was prepared, the PC was fixed in sealed quartz cuvette with enough liquid ethanol to produce a saturated ethanol gas circumstance. After 30 s delay, the transmittance spectrum in wavelength between 400 nm and 800 nm was recorded repeatedly. The two adjacent transmittance spectra have a 90 s measurement time interval.

### Characterization

The morphologies of the samples were observed by a Field Emission Scanning Electron microscopy (FE-SEM, Sirion 200), and the transmittance spectra were measured by spectrophotometer (CARY 5E) with incident light perpendicular to the sample surface.

## Author Contributions

G.T.F. and G.L.S. designed the experiments. G.L.S. and Y.Z. conducted simulations. G.L.S., G.T.F. and Y.Z. analysed data. G.T.F., G.L.S., Y.Z., P.Y., S.H.X., H.M.O. and L.D.Z. discussed the results and contributed to the writing of the manuscript.

## Supplementary Material

Supplementary Information Fano resonance in anodic aluminum oxide based photonic crystals
