# Supplementary material for: Fano resonance in anodic aluminum oxide based photonic crystals
Source: Sci Rep. 2014 Jan 8;4:3601. doi: 10.1038/srep03601 (PMC3884230; doi:10.1038/srep03601)
Supplement: Supplementary Information — Fano resonance in anodic aluminum oxide based photonic crystals [file srep03601-s1.doc]

Fano resonance in anodic aluminum oxide based photonic crystals

Guo Liang Shang^1^, Guang Tao Fei^1*^, Yao Zhang^1,2^, Peng Yan^1^, Shao Hui Xu^1^, Hao Miao Ouyang^1^ & Li De Zhang^1^

1 Key Laboratory of Materials Physics and Anhui Key Laboratory of Nanomaterials and Nanostructures, Institute of Solid State Physics, Hefei Institutes of Physical Science, Chinese Academy of Sciences, P. O. Box 1129, Hefei, 230031, P. R. China

2 Hefei National Laboratory for Physical Sciences at the Microscale, University of Science and Technology of China, Hefei, Anhui 230026, P. R. China

*Corresponding author: E-mail: gtfei@issp.ac.cn

**Supplementary Information:**

During the second oxidation, the compensation voltage mode was applied.^33^ In detail, in the first period of the voltage waveform, the voltage increases from *V_L_* (=23 V) to *V_H_* (=53 V) by a quarter of sinusoidal wave within *t_a_*=30 s and then linearly decreases to *V_L_* within *t_b_*=3 min. From the second period, a compensation voltage was introduced in order to overcome the chemical corrosion and the ion exchange rate decrease from the long term oxidation.^33,34^ The voltage value of each period overall increases with a *V_increase_* of 0.055 V compared to the previous period. All the second oxidations were controlled by computer and carried out in a water tank with constant temperature of 16 °C. Finally, the remaining aluminum was removed by etched in saturated CuCl_2_ solution, and the barrier layer was removed with 3 wt.% phosphoric acid at 40 °C.

The anodic oxidations mode will generate a periodical porous layered structure associated to the applied voltage. In time *t_a_*, the stem channel are formed, and in the following time *t_b_*, the stem channels grow. When the applied voltage decreases to 1/√2 times of the maximum voltage (*V_H_*), the stem channels become to two channels. In the following time of *t_b_*, the branched channels grow. Such periodic waveform of the voltage produces a corresponding periodic structure composed of stem channel layers and branched channel layers alternated along the pore growing direction.


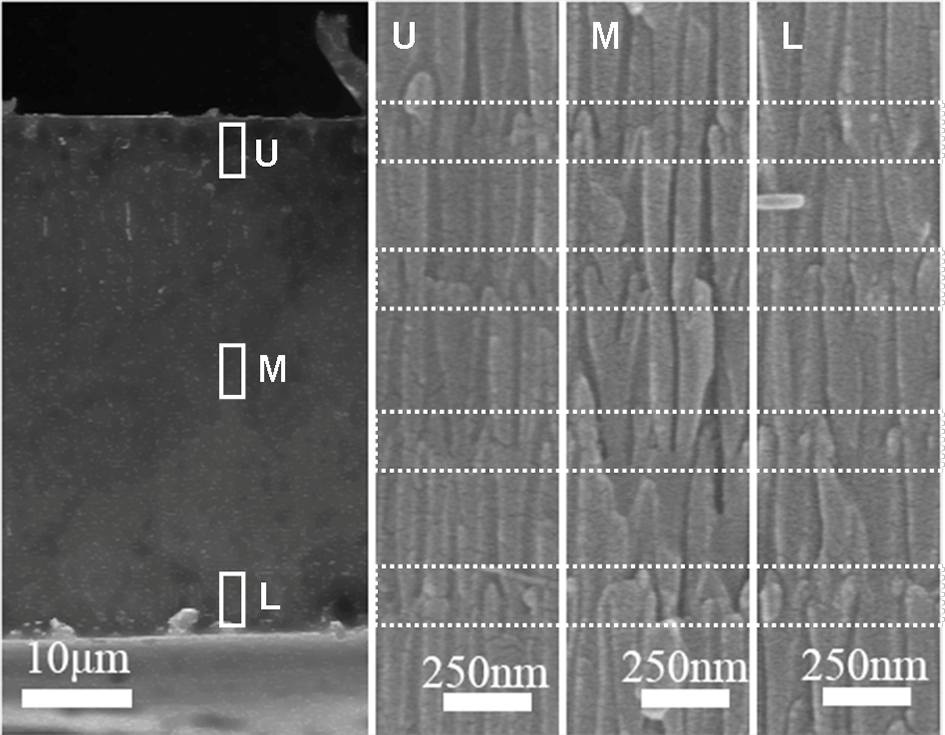


**Figure S1 | Cross-sectional SEM images of the as-prepared sample.** U, M, L stands for upper, middle and lower part of the sample, respectively. White dash lines are the interfaces between stem channel layer and branched channel layer.

Uniform structure of the AAO based PC was prepared by compensation voltage method (Fig. S1).^33^ It can be found that the as-prepared PC is about 47 μm thick, and the identifier U, M, L stands for enlarged images of upper, middle and lower parts of the PC. As we can see from the enlarged images, the PC has a nearly uniform pore structure from upper to lower part, and this uniform structure will beneficial for the generation of well PBG.

**Supplementary References:**

33 Shang, G. L. *et al.* Preparation of narrow photonic bandgaps located in near infrared region and their applications on ethanol gas sensing. *J. Mater. Chem. C*, **1**, 5285-5291 (2013).

34 Shang, G. L. *et al.* Preparation of the very uniform pore diameter of anodic alumina oxidation by voltage compensation mode. *Mater. Lett.*, **110**, 156-159 (2013).
